# Natural variation in yolk fatty acids, but not androgens, predicts offspring fitness in a wild bird

**DOI:** 10.1186/s12983-021-00422-z

**Published:** 2021-08-05

**Authors:** Lucia Mentesana, Martin N. Andersson, Stefania Casagrande, Wolfgang Goymann, Caroline Isaksson, Michaela Hau

**Affiliations:** 1 Max Planck Institute for Ornithology, Eberhard-Gwinner-Str., 82319 Seewiesen, Germany; 2 Department of Biology, Lund University, Sölvegatan 37, 223 62 Lund, Sweden; 3 University of Konstanz, Universitätsstraße 10, 78464 Konstanz, Germany

**Keywords:** Maternal effects, Fitness, Phenotypic variance, Steroid hormones, Antioxidants, Fatty acids

## Abstract

**Background:**

In egg-laying animals, mothers can influence the developmental environment and thus the phenotype of their offspring by secreting various substances into the egg yolk. In birds, recent studies have demonstrated that different yolk substances can interactively affect offspring phenotype, but the implications of such effects for offspring fitness and phenotype in natural populations have remained unclear. We measured natural variation in the content of 31 yolk components known to shape offspring phenotypes including steroid hormones, antioxidants and fatty acids in eggs of free-living great tits (*Parus major*) during two breeding seasons. We tested for relationships between yolk component groupings and offspring fitness and phenotypes.

**Results:**

Variation in hatchling and fledgling numbers was primarily explained by yolk fatty acids (including saturated, mono- and polyunsaturated fatty acids) - but not by androgen hormones and carotenoids, components previously considered to be major determinants of offspring phenotype. Fatty acids were also better predictors of variation in nestling oxidative status and size than androgens and carotenoids.

**Conclusions:**

Our results suggest that fatty acids are important yolk substances that contribute to shaping offspring fitness and phenotype in free-living populations. Since polyunsaturated fatty acids cannot be produced de novo by the mother, but have to be obtained from the diet, these findings highlight potential mechanisms (e.g., weather, habitat quality, foraging ability) through which environmental variation may shape maternal effects and consequences for offspring. Our study represents an important first step towards unraveling interactive effects of multiple yolk substances on offspring fitness and phenotypes in free-living populations. It provides the basis for future experiments that will establish the pathways by which yolk components, singly and/or interactively, mediate maternal effects in natural populations.

**Supplementary Information:**

The online version contains supplementary material available at 10.1186/s12983-021-00422-z.

## Background

Understanding the causes of phenotypic variation is a major goal in evolutionary biology. Maternal effects are now recognized as important factors that contribute to phenotypic variation (meta-analysis by [1]), although the magnitude and direction of maternal effects is still under debate [2, 3]. Mothers shape offspring phenotype not only through the genes they pass on to their offspring but also by influencing the environment their offspring experience during development [4–6]. In egg-laying species, mothers can influence the embryonic environment by allocating different resources to the yolk, including hormones, antioxidants and fatty acids, among other components [7–14]. The importance of some of these maternally transmitted compounds for phenotypic traits has been documented in various taxa (e.g., [9, 15, 16]). In birds, yolk steroid hormones, such as androgens and glucocorticoids, can influence offspring growth, competitive ability and survival (e.g., *Ficedula albicollis*; [17, 18] and reviewed by [19]), but they can also increase chick susceptibility to oxidative stress by increasing the production of reactive oxygen species or by impairing antioxidant defenses (e.g., *Gallus gallus*; [20, 21]). Maternally derived antioxidants, such as carotenoids or vitamin E, can promote growth (reviewed by [22]) and limit the negative consequences of increased oxidative stress by scavenging the reactive oxygen species produced during growth (e.g., *Gallus gallus, Larus michahellis, Parus major*; [23–25]). Fatty acids are an important source of energy; they can enhance the general viability and proper development of both embryos and nestlings (reviewed by [26, 27]). In particular, polyunsaturated fatty acids (PUFAs) are essential components for the formation of cell membranes, heart function and brain development (e.g., [28]). At the same time, PUFAs are susceptible to damage from lipid peroxidation by reactive oxygen species that are generated as by-products of offspring metabolism [29]. Thus far, the evidence supporting a relevance of fatty acids for the phenotypic development of embryos and nestlings has come mainly from studies on poultry or captive birds (reviewed by [30]), in which environmental conditions are typically designed to be benign. Yolk fatty acids have been shown to vary with environmental conditions in free-living birds (*Cyanistes caeruleus, Parus major* [31, 32]), but it remains unclear whether they play a role in shaping offspring fitness and phenotype in natural populations that experience frequent fluctuations in food availability and weather conditions.

Maternal effects through egg deposition are often studied by measuring the effect of single yolk components (in particular androgens) or groups of related components (e.g., steroid hormones) on offspring fitness and phenotypic traits (reviewed by [33, 34]). This approach has been pivotal for advancing our knowledge of how mothers affect offspring phenotype. However, maternal effects are by nature multivariate (reviewed by [33, 34]), with different groups of yolk components influencing similar nestling traits, i.e., by having interactive effects on offspring phenotype [35–37]. For instance, hatchling mass was reduced and oxidative stress increased in Japanese quails (*Coturnix japonica*) hatching from eggs injected with either testosterone or carotenoids [35]. But when both components were administered together, neither hatchling mass nor oxidative stress were affected. Similar compensatory effects were found in yellow-legged gulls (*Larus michahellis*) after eggs were simultaneously injected with corticosterone and vitamin E [36, 37]. Such studies suggest that the consequences of maternal effects on offspring phenotype known from studies that focus on single components might actually be absent, weakened or potentiated if the presence and actions of other components are also considered.

Here we studied the relationship of multi-substance yolk composition with nestling fitness and phenotype in a wild population of great tits over two years. This observational study is powerful in assessing natural variation in yolk composition and offspring fitness and phenotypes, and provides an important basis for subsequent experimental work. We measured the concentrations of 31 yolk components in the fourth egg from 69 clutches, including 4 steroid hormones, 3 antioxidants and 24 fatty acids. We grouped yolk components using a principal component analysis, although a discussion of the associations is beyond the scope of this manuscript. We then analysed the relationships between these groupings of yolk components and offspring fitness proxies, such as hatchling and fledgling number. We also determined links between yolk components and phenotypic traits like growth (i.e., body mass and tarsus length) and oxidative status of the nestlings from a given nest. The oxidative status of an individual is defined as the concentrations of pro-oxidants (i.e., reactive oxygen species) and antioxidants (i.e., non-enzymatic and enzymatic compounds) present in cells and tissues (reviewed by [38]). A change in any of the molecular components of the oxidative system in favor of pro-oxidants can damage crucial molecules, such as lipids, proteins and DNA (reviewed by [39]), with potential fitness consequences (e.g., [40] and meta-analysis by [41]). Nestling oxidative status has therefore been proposed to mediate their survival and health (e.g., *Fregata magnificens* [42]). Because the strength of maternal effects declines throughout offspring ontogeny (meta-analysis by [1]) and because the nestling oxidative status can change during the growth period (e.g., antioxidant defences; *Taeniopygia guttata*; [43]), we measured chick phenotypic traits at two time points: before and after nestlings reached exponential growth (i.e., 6 and 12 days post-hatching, respectively).

## Results

### Covariation of yolk components

Using PCA, we identified the relationships among all 31 yolk components (Additional file 1). PC1 was negatively associated with vitamin E, one specific monounsaturated fatty acid (MUFA; 20:1n − 9) and all ω − 6 PUFAs (16:2n − 6, 18:2n − 6, two 18:3n − 6, 20:2n − 6, 20:3n − 6, 20:4n − 6, 22:4n − 6). PC2 was positively associated with four saturated fatty acids (SFAs; 15:0, 16:0, 17:0 and 18:0), all but one MUFA (18:1n − 9, 16:1n − 9, 16:1n − 7, 18:1n − 7) and all ω − 3 PUFAs (18:3n − 3, 20:5n − 3, 22:5n − 3, 22:6n − 3). Androgens (androstenedione, 5α-dihydrotestosterone and testosterone) and carotenoids (lutein and zeaxanthin) loaded positively onto PC3 whereas two MUFAs (18:1n − 9, 16:1n − 9) loaded negatively onto PC3. To simplify the description of the results and the visualization of the figures, hereafter all PCs are discussed in terms of positive loadings (i.e., we transformed PC1 into positive values). Also, as almost all MUFAs were included in PC2, whereas only one or two were present in PC1 and PC3, and the effects of different groups fatty acids are more relevant to our research question rather than specific fatty acids, the effects of MUFAs on nestling phenotype are discussed primarily based on results for PC2. Corticosterone was the only yolk component for which only low values loaded on the PCs. Corticosterone concentration is the least repeatable trait in yolk (*R* ~ 0.18; [10]), thus yolk corticosterone concentrations in the fourth egg do not reliably predict concentrations in the other eggs of the same clutch. Therefore, we decided to forego further analysis of corticosterone in this study.

### Egg components and fitness proxies

**Fig. 1:**
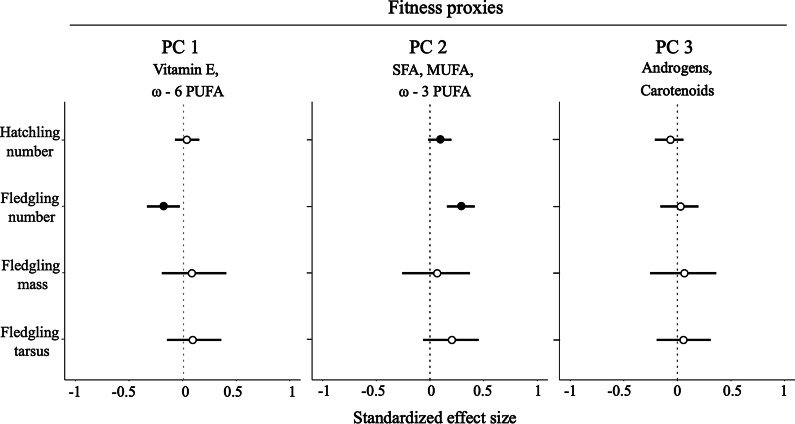
Relationships between egg yolk components and fitness proxies in great tit nestlings. Yolk components are grouped based on a principal component analysis. PC1 was mainly represented by high concentrations of vitamin E (α-tocopherol) and ω-6 polyunsaturated fatty acids (PUFAs), PC2 by high concentrations of saturated fatty acids (SFAs), mono-unsaturated fatty acids (MUFAs) and ω-3 PUFAs, and PC3 by high concentrations of androgens (androstenedione, 5α-dihydrotestosterone and testosterone) and carotenoids (lutein and zeaxanthin; Additional file 1). Shown are the standardized effect sizes with their corresponding 95% credible intervals (CrIs). Filled circles indicate statistically meaningful support (i.e., if the mean difference between compared estimates is higher than 0.95) for the effect of a set of yolk components on fitness proxies

Nests with eggs that had higher SFA, MUFA and ω − 3 PUFA concentrations had higher numbers of hatchlings and fledglings (Fig. 1, Additional file 2) than nests containing eggs with lower concentrations of these fatty acids. Furthermore, fledgling number was lowest in nests with eggs with increased concentrations of vitamin E and ω − 6 PUFAs. Neither androgens nor carotenoids explained fitness proxies. Fledgling mass and tarsus length were not related to any yolk component.

### Egg components and phenotypic traits

**Fig. 2:**
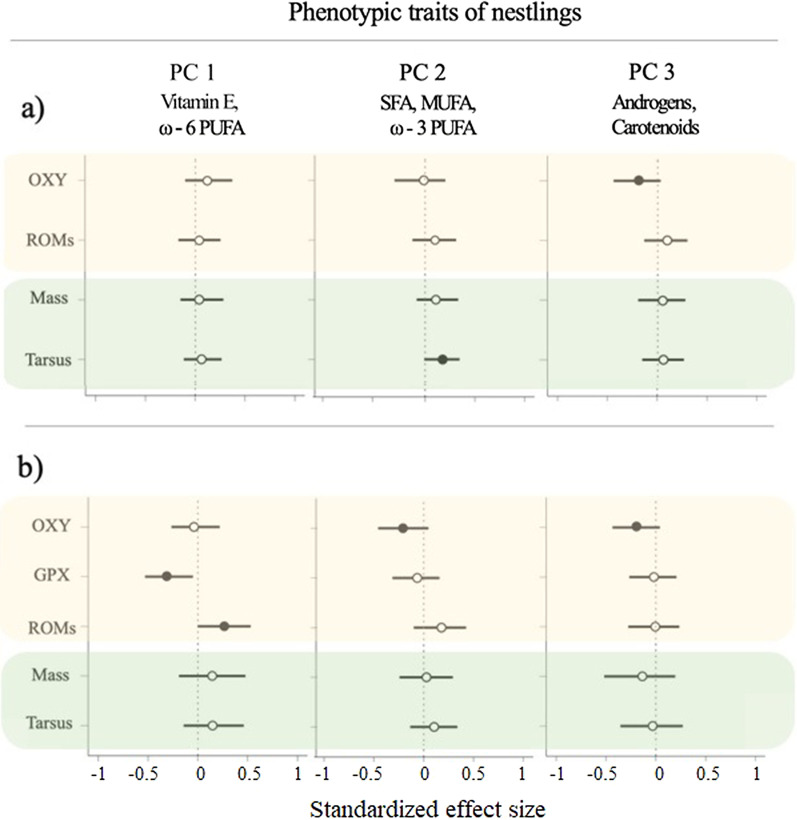
Relationships between egg yolk components and physiological traits in great tit nestlings on days (**a**) 6 and (**b**) 12. Yolk components are grouped based on a principal component analysis. PC1 was mainly represented by high concentrations of vitamin E (α-tocopherol) and ω-6 polyunsaturated fatty acids (PUFAs), PC2 by high concentrations of saturated fatty acids (SFAs), mono-unsaturated fatty acids (MUFAs) and ω-3 PUFAs, and PC3 by high concentrations of androgens (androstenedione, 5α-dihydrotestosterone and testosterone) and carotenoids (lutein and zeaxanthin; Additional file 1). Nestling oxidative status and body condition are indicated with yellow and green shading, respectively. Shown are the standardized effect sizes with their corresponding 95% credible intervals (CrIs). Filled circles indicate statistically meaningful support (i.e., if the mean difference between compared estimates is higher than 0.95) for the effect of a set of yolk components on physiological traits. Oxidative status parameters: OXY, non-enzymatic antioxidants present in plasma; GPX, enzymatic antioxidant present in red blood cells; ROMs, reactive oxygen metabolites present in plasma

On day 6, nestlings that hatched from clutches with eggs containing high concentrations of SFAs, MUFAs and ω − 3 PUFAs had longer tarsi than nestlings that hatched from nests with eggs containing low concentrations of these fatty acids (Fig. 2a, Additional file 3). Yolk androgen and carotenoid concentrations were negatively related to nestling non-enzymatic antioxidant concentrations (i.e., OXY), whereas mass was not associated with any group of components. On day 12, the oxidative status of great tit nestlings was explained by all yolk component groups, but in different ways (Fig. 2b, Additional file 4). In particular, nestlings hatching from nests with eggs that had high concentrations of vitamin E and ω − 6 PUFAs had high concentrations of reactive oxygen metabolites (i.e., ROMs) in plasma and low levels of enzymatic antioxidant glutathione peroxidase (i.e., GPX) in red blood cells. Plasma OXY concentrations were negatively related to high concentrations of SFAs, MUFAs and ω − 3 PUFAs, and, as on day 6, also to high concentrations of androgens and carotenoids. Body mass and tarsus length were not related to any group of yolk components.

## Discussion

Natural variation in fatty acid content was the only group of yolk components that explained variation in nestling fitness proxies in a free-living population of great tits. In contrast, androgens and carotenoids—yolk components that were previously shown to affect chick development and fitness (e.g., [17, 18] and reviews by [19, 22])—did not account for variation in fitness. Furthermore, the two yolk component groupings that included fatty acids were related to more aspects of the physiological phenotype of the nestlings (size and oxidative status) than androgens and carotenoids (only one measure of oxidative status). Hence, natural variation in the fatty acid content of yolk may play a major role in determining the fitness and phenotype of offspring in a wild bird population.

### Egg components and fitness proxies

In our study, yolk fatty acids were related to both hatchling and fledgling numbers. Yolk fatty acids have been reported to promote fitness in captive invertebrates (e.g., *Penaeus chinensis*, *Acartia erythrae*a; [44, 45]) and vertebrates (e.g., birds: *Gallus gallus* [46]), but so far ours is the first study to indicate a relationship with fitness-related traits in a free-living population exposed to natural variation in environmental conditions.

In general, the importance of yolk fatty acid composition for hatchling and fledgling numbers might depend on the female’s diet during egg laying. During the laying period, great tits change from feeding on seeds to predominantly feeding on caterpillars [47, 48]. Caterpillars are rich in SFAs, ω − 3 PUFAs (i.e., α-linolenic acid) and antioxidants [49–51], i.e., in substances that are thought to be beneficial for offspring fitness. However, if caterpillars are scarce because of low ambient temperatures or poor habitat quality, adult great tits rely on other invertebrates like arachnids [52], which are richer in ω − 6 PUFAs [50, 51]. Whilst this dietary flexibility allows parents to maintain food intake, it may compromise the optimal balance of nutrients that females can deposit into their eggs. Females can store and mobilize all fatty acids from internal stores to plasma (reviewed by [53]) and then to the yolk. However, since the PUFA and antioxidant composition of females is strongly linked to their diet (e.g., *Parus major*; [54]), variation in maternal food resources may directly affect hatchling and fledgling numbers.

Hatchling and fledgling numbers were highest in the nests of great tit mothers that laid eggs whose overall concentrations of SFAs, MUFAs and ω − 3 PUFAs were high (Fig. 1). SFAs and MUFAs, which are the most abundant fatty acids in the yolk (e.g., [10, 32]), can be endogenously produced (i.e., they are not essential fatty acids; reviewed by [27]), and represent an important source of energy. ω − 3 PUFAs are particularly important in early life stages when nestlings are developing and growing fast, and can enhance bone formation and immune competence (in birds e.g., [55–57]). Hence, eggs containing high concentrations of SFAs, MUFAs and ω − 3 PUFAs might have provided the embryo with both the energy and essential components that enhance development, and thereby may have boosted the number of chicks that hatched in this study. These fatty acids may have promoted early growth, as nestlings in our study that emerged from nests that had such eggs had longer tarsi on day 6 (Fig. 2a, Additional file 3). In our population, long tarsi are a good proxy for survival during the nestling phase (see Additional file 5), thus probably also contributing to increased fledgling numbers. Alternatively, eggs containing high concentrations of SFAs, MUFAs and ω − 3 PUFAs could have also had an overall high lipid content (reviewed by [26]). Therefore, in the future it will be important to test whether hatchling and fledgling numbers are higher in eggs that contain high concentrations of specific fatty acid groups or have an overall high lipid content.

By contrast, high concentrations of one class of fatty acids, ω − 6 PUFAs, and that of the antioxidant vitamin E were negatively related to fledgling number (Fig. 1). We offer two non-mutually exclusive explanations for this finding. Firstly, in great tits one would expect to observe an inverse relationship between the two types of ω-PUFAs in the yolk given that ω − 3 PUFAs and ω − 6 PUFAs are obtained from different food sources (see above; [32]). Hence, nestlings developing from nests with eggs containing high concentrations of ω − 6 PUFAs and vitamin E might have had lower concentrations of other resources that might have boosted fledging success. Secondly, poultry studies indicate that a ratio of high ω − 6 PUFA concentrations relative to those of ω − 3 PUFAs is associated with the formation of pro-inflammatory eicosanoids (i.e., mediators of inflammation), whilst the inverse ratio is associated with the formation of anti-inflammatory eicosanoids (e.g., [56, 57]). If chicks needed to mount inflammatory processes during the nestling phase, those chicks developing from clutches with a ratio of high ω − 6 versus ω − 3 PUFAs in their yolk might have shown strong inflammatory responses that could have lowered their fledging success.

In the current study, offspring fitness proxies were not related to yolk androgen and carotenoid concentrations, in contrast to several publications showing an impact of these yolk components on early development and survival in birds (e.g., [17, 18] and reviews by [19, 22]). Here we offer three non-mutually exclusive explanations for this result, which could be tested in subsequent experiments. On the one hand, and unlike many previous studies that focused on one group of yolk components (e.g., androgens, e.g., [17]), we simultaneously measured three different groups of yolk components, and some, like fatty acids, explained more variation in offspring phenotype and fitness than androgens and carotenoids. Thus, a potentially small effect of androgens and/or carotenoids might have been masked by a stronger influence of fatty acids. On the other hand, our study design imposed two limitations. Firstly, we analyzed the relationship between fitness and androgens/carotenoids by using PC3. However, this PC3 also included two MUFAs, which might have obscured the link with androgens and carotenoids—although we consider this possibility unlikely. Secondly, to be able to assess offspring fitness and phenotypes, we collected the middle egg as a representative for each clutch and allowed its siblings to develop normally. This technique is commonly used to study the adaptive value of maternal yolk deposition (in this species see [58, 59]; see also e.g., *Ficedula albicollis* [18, 60]) because alternative methods, like taking a biopsy of the yolk from each egg, can result in a substantial reduction of offspring fitness (e.g., hatching success; *Troglodytes aedon*, [61]) while effects on offspring phenotypes are unclear. In our population of free-living great tits, the concentrations of many yolk components exhibit a considerable repeatability (androgens *R* = 0.30–0.64 and carotenoids *R* = 0.38–0.39; [10]), suggesting that females are relatively consistent in their deposition of yolk components within a clutch (see also [62]). Nevertheless, despite the repeatability there also remains a considerable amount of residual variance (~ 36–70%; (1 − *R*)), which likely includes plasticity along the laying sequence in the concentrations of yolk components that mothers deposit [10]. For those reasons, the lack of an association between yolk androgen and carotenoid concentrations with fitness traits requires further investigation in a multi-component study.

### Egg components and phenotypic traits

Our study indicates that maternal yolk deposition also shapes offspring phenotypic variation, but that its influence varies across traits and developmental stages (Fig. 2a, b). In particular, yolk substances showed a stronger link with physiological traits considered to be indicators of nestling health (oxidative status) than with morphological traits. In fact, only one morphological trait (tarsus length) was explained by yolk content and only on day 6, before nestlings reached exponential growth.

Fatty acids, antioxidants and androgens were all associated with the oxidative status of nestlings, albeit via different components of the redox system (Fig. 2a, b). High yolk concentrations of ω − 6 PUFA and vitamin E, which are components negatively related to fledgling number, were associated with a low oxidative status of nestlings on day 12 (i.e., nestlings had low enzymatic antioxidant concentrations in red blood cells and high concentrations of oxidative damage in plasma). Conversely, high concentrations of SFAs, MUFAs and ω − 3 PUFAs, yolk components positively associated with hatchling and fledgling numbers, were linked to low concentrations of non-enzymatic antioxidants in plasma of nestlings on day 12 (but were not associated with nestling oxidative damage). Lastly, high yolk concentrations of androgens and carotenoids were also related to low concentrations of non-enzymatic antioxidants in nestlings on days 6 and 12. These results lead us to speculate that the concentrations of yolk fatty acids influence the oxidative status of the nestlings on day 12, thus also explaining the differences observed in fledgling numbers. However, this seems not to be the case in our population given that the majority of the nestlings recorded on day 12 (N = 160) survived to day 15 (N = 153).

In our study, maternal yolk deposition predicted fewer aspects of the nestlings’ oxidative status on day 6 compared to day 12 (Fig. 2). These results are in contradiction with the hypothesis that the strength of maternal effects declines throughout offspring development (e.g., [63]). However, our results could be explained if the development of the great tit nestling antioxidant system occurs during similar ontogenetic times as in poultry chicks. In poultry, carotenoids and vitamin E are transferred from the egg yolk to the chick’s tissues during the last week of incubation. Soon after hatching, nestlings are not yet able to effectively assimilate antioxidants from the diet and their antioxidant system is mainly determined by the concentration of yolk antioxidants deposited by the mother (reviewed by [64]). One to two weeks after hatching, a chick’s plasma concentrations of carotenoids and vitamin E decrease dramatically (reviewed by [64, 65]), and subsequently the antioxidant protection of a nestling is increasingly determined by its own production of enzymatic antioxidants like GPX (reviewed by [65]). In free-living populations, the temporal dynamics of chick antioxidant protection provided by maternal yolk resources versus the chick’s own antioxidant production remains to be studied. But if a similar temporal pattern of antioxidant protection as in poultry chicks occurs in free-living great tit nestlings, both a decrease in maternally derived antioxidants and an increase in metabolic demands during the nestling period could explain the stronger relationship between maternal yolk hormones and the nestlings’ oxidative status observed on day 12 compared to day 6. This could especially be the case for nestlings developing from clutches where the fourth egg had high concentrations of ω − 6 PUFA and vitamin E, since the former component is susceptible to damage from lipid peroxidation by reactive oxygen species that are generated as by-products of offspring metabolism.

## Conclusions

Our study suggests a novel link between natural variation in yolk fatty acid content and offspring fitness and phenotype in a wild bird population. It also indicates that groupings of yolk components can be related to fitness and phenotypic traits of great tit nestlings in different ways. This finding, together with previous studies showing that substances are co-secreted into the yolk (e.g., [66]), supports the idea that the consequences of maternal yolk deposition for the offspring should be addressed by simultaneously studying several components rather than single yolk components [34]. Our study represents the first step towards an integrative understanding of how steroid hormones, antioxidants and fatty acids may jointly shape offspring development. Carefully designed experimental studies, in which egg components are being manipulated both in isolation and simultaneously, are now needed to test for the causal effect that each component—as well as in interaction with other yolk substances—have on the offspring.

Variation in offspring fitness proxies was explained by some yolk components that mothers have to acquire from their diet (i.e., vitamin E and both PUFA classes). This suggests that offspring fitness also depends on the female’s access to, or ability to exploit, high-quality food sources [30]. A female’s diet can be influenced by both genetic and environmental factors and it will now be important to determine the relative contributions of these two variables to yolk composition. Further, if hatchling or fledgling numbers were mainly related to concentrations of essential (dietary) yolk components, offspring fitness would greatly rely on the ability of females to find food. In this scenario fluctuations in food abundance, either because of natural variation in weather and habitat quality or human modification of the environment, could have direct fitness consequences for the offspring. Alternatively, if fitness traits were more strongly influenced by non-essential yolk components (i.e., SFA or MUFAs), offspring fitness would depend on the physiological capacity of females to produce and transfer these components into the yolk. Also, it is worth noting that in this study we cannot fully exclude the possibility that nestling fitness proxies and phenotypes could result from differences in habitat quality among breeding pairs that influenced the quality of the food delivered by the parents to their young. Future experimental studies should test to what extent differences in nestling fitness proxies and phenotypes are related solely to maternal yolk components versus nestling food quality.

By collecting the fourth egg, we determined the relationship between the average deposition of yolk components and offspring fitness and phenotype. Future studies should investigate how other sources of variation in female yolk deposition (i.e., the plasticity of deposition along the laying sequence and the covariance between average deposition and plasticity; e.g., [10, 67]) shape offspring traits.

## Methods

### Study species and field site

We studied a nest-box population of great tits from April to July in both 2015 and 2016 in the Dellinger Buchet, in Southern Germany (Bavaria; 48° 03′ N, 11° 13′ E, 620 m above sea level), a forest with a mosaic of deciduous and coniferous trees. Female great tits generally lay one egg each day. In our population, mean clutch size is (mean ± SD) 8.45 ± 1.13 eggs, incubation starts after the last egg is laid and lasts approximately 14 days (mean ± SD = 14.07 ± 2.77), and nestlings spend around 20 days in the nest (mean ± SD = 20.60 ± 1.43; our own unpubl. data). In this study, we only considered first clutches.

### Nest monitoring and egg collection

We visited nests every second day from the beginning of the breeding season onwards. Once egg laying started, we visited nests every day and marked eggs with a pencil to identify the laying order. Great tit females vary substantially in the mean allocation of yolk components and in their plasticity along the laying sequence (e.g., [10, 62]); yet, in our population, we previously reported that yolk components show medium repeatabilities for androgens (*R* = 0.30–0.64) and antioxidants (*R* = 0.36–0.39), and medium–high repeatabilities for fatty acids (*R* > 0.5 for SFAs and MUFAs and *R* > 0.9 for PUFAs; [10]). These repeatability estimates suggest that the concentrations of yolk components in eggs laid by the same female are more similar to each other than to eggs laid by other females. These medium–high repeatabilities also suggest that the middle egg of each clutch represents the average yolk content for a given clutch. In the present study, we therefore collected the fourth egg because it represents, on average, the middle egg of a great tit clutch (for a similar approach in this species see [58, 59]. We collected eggs between 8:00 and 13:00 h on the day they were laid and replaced them with a dummy egg. All collected eggs from a total of 69 clutches were laid by different females, except for two focal females that were included in both years. On the day of collection, we weighed and opened each freshly laid egg in the laboratory and separated the yolk from the albumen by rolling it on a piece of paper following [68]. We then homogenized the yolk by mixing it with an equal amount of distilled water (1 µl per mg of yolk) and stored it at − 80 °C until further analysis.

### Nestling growth monitoring and blood sampling

We checked nests until clutch completion, as identified by the absence of freshly laid eggs and incubation behaviour of females. Two days prior to expected hatching, we monitored each nest closely to record the hatching date (day 0 = the day the first hatchling was observed). On day 1, we identified each nestling individually by clipping several down feathers, and we recorded its body mass (to the nearest 0.1 g). On day 6 or 7 (hereafter referred to as day 6) and on day 12 or 13 after hatching (hereafter referred to as day 12), we measured nestlings’ oxidative status. We selected these two periods because they are just before and after great tit nestlings reach exponential growth (i.e., on day 9–10 post-hatching; [69]), and because nestlings are large enough to collect the minimum blood volumes required to analyse their oxidative status. In particular, on day 6 we collected a small blood sample (~ 20 μl) from the brachial vein with heparinized capillaries to determine nestling oxidative status. We sampled all nestlings (N = 182) from the same nest (N = 51) within 15 min of disturbance (mean ± SD = 12.72 ± 6.93), again recording body mass and tarsus length (to the nearest 0.1 mm). On day 12, we collected the second blood sample (~ 80 μl) to determine each nestling’s oxidative status. We took samples within 3 min of disturbance (mean ± SD = 1.86 ± 0.73) from two or three randomly selected chicks (N = 96) per nest (N = 36), and recorded body mass and tarsus length. We then fitted each nestling with a numbered aluminum ring. On day 15, we recorded final body mass and tarsus length, and fledging was monitored until all young had left the nest. Finally, we also recorded the environmental conditions experienced by great tits during the incubation and nestling periods (see Additional file 6).

### Yolk analyses

In total, we measured 31 yolk components (Additional file 2). The equipment, methods and further assay details used to measure each yolk component are described in the Additional file 6 (see also [10]). Briefly, we separated steroid hormones (androstenedione, 5α-dihydrotestosterone, testosterone, and corticosterone) via diatomaceous earth columns. For this, we followed the method described by [70], modified by [71] with additional adjustments for the measurement of egg yolk following [72]. We quantified androgen concentrations using radio-immunoassays, assaying each sample in duplicate and correcting hormone concentration of each sample for its’ individual extraction efficiency. All samples were analysed in two assays. Intra-assay coefficients of variation, as determined from the positive controls containing stripped chicken plasma with a known quantity of hormone added, were: androstenedione = 9.6%, 5α-dihydrotestosterone = 14.9% and testosterone = 23.8%. The inter-assay coefficients of variation, as determined from the first positive control of each assay, were: androstenedione = 13.3%, 5α-dihydrotestosterone = 13.6% and testosterone = 22.3%. We determined corticosterone concentrations using enzyme immunoassays (lot numbers: 12041402D and 04281702, Enzo Life Sciences, Germany). We assayed each sample in duplicate and distributed samples across five assays. The intra-assay coefficients of variation, also determined from stripped chicken plasma with a known quantity of corticosterone added, were 6.2, 11.4, 19, 7 and 12.3%, and the inter-assay coefficient of variation was 10.8%.

We extracted and then quantified antioxidants (lutein, zeaxanthin and vitamin E) by high-performance liquid chromatography (HPLC) following [10] (for details see Additional file 6). We calculated antioxidant concentrations from standard curves made for lutein, zeaxanthin and vitamin E (α-tocopherol) along with corrections for their respective internal standards. Of the 69 eggs analysed, data on the concentration of lutein and zeaxanthin were missing for one and two eggs, respectively. We assigned the value of the average population of each antioxidant to those eggs with missing values [73].

We extracted and subsequently analysed fatty acids (saturated fatty acids: SFAs; mono-unsaturated fatty acids: MUFAs; ω − 3 and ω − 6 polyunsaturated fatty acids: PUFAs) using gas chromatography-mass spectrometry (GC–MS), according to previously established methods [10, 74] (for details see also Additional file 6). We quantified fatty acid concentrations based on the peak area of the internal standard (methyl *cis*-10-heptadecenoate; 16.65 µg added to each sample).

### Oxidative biomarkers measurements

On days 6 and 12, we determined the concentrations of antioxidants and oxidative damage in nestling plasma (details on the equipment and methods used are described in Additional file 6). Briefly, we measured OXY (expressed as mM HOCl neutralized) using the OXY-Adsorbent test (Diacron International SRL, Grosseto, Italy) following [75], and oxidative damage (ROMs: produced by the oxidation of lipids, proteins and nucleic acids, and are expressed as mM H2O2 equivalents) using the d-ROMs test kit (Diacron International SRL, Grosseto, Italy) following [75]. We assayed each sample in duplicate and samples were analysed across 21 plates. Inter-assay coefficients of variation, as determined from the calibrators (OXY) or from known standards (ROMs), were 5.1% and 2.8% for OXY and ROMs respectively. On day 12, we additionally measured the enzymatic activity of GPX (expressed as U/ml) in red blood cells using the Ransel assay (Randox Laboratories, Germany) following [76].

### Statistical analysis

To group yolk components, we ran a principal component analysis (PCA), including all 31 yolk components analysed (for further details on this analysis see Additional file 6). The three principal components (PC) identified accounted for 58% of the cumulative variance in our data (Additional file 1). The mean values of the principal components differed between years (Additional file 7). To account for year differences, we standardized the loadings because ‘year’ could not be included as a random (we only had two levels) or a fixed factor (limited sample size). Thus, for each nest we first subtracted and then divided the loading of each principal component by the mean value of that component in the corresponding year. This method allowed us to assess the importance of each yolk component irrespective of year differences [77].

To study the relationship between yolk components in the fourth egg of clutches and fitness proxies for these clutches, we first ran two generalized linear models (we initially ran two generalized linear mixed effect models by including ‘female identity’ as a random factor, but since this factor did not explain any variance, we excluded it from all final models). Hatchling and fledgling number were fitted as response variables, PC1, PC2, PC3, date of egg collection and clutch size were included as covariates (two clutches were excluded from analyses; see Additional file 6). Body mass at fledging is a key predictor of post-fledging survival in great tits [78]. As other proxies of fitness, we therefore assessed the relationship between yolk components and nestling mass and tarsus length on day 15. These morphological variables were studied in separate models because yolk components can influence structural body size (i.e., tarsus length) independently of mass (e.g., [55]). Because of sample size constraints and to reduce the number of explanatory variables, we fitted the residuals of a linear regression between these two estimates of body condition and clutch size as response variables in two separate linear mixed-effect models. PC1, PC2, PC3 and capture date were included as covariates in the model, and nest ID was fitted as a random factor. Environmental variables were initially included in the analyses, but since they did not influence the response variable we excluded them from all final models (for details see Additional file 6).

To study the relationship between egg components and the physiological condition of individual nestlings from a given brood, we ran linear mixed-effect models. We fitted OXY, GPX (only for day 12), ROMs, nestling mass and tarsus length (corrected for brood size) as response variables. We included PC1, PC2, PC3 and date of egg collection as covariates, and nest identification as a random factor. We initially included other covariates in the model based on their biological relevance to the study question (see Additional file 6), but because of sample size constraints, only variables that had an effect on the response variables were retained in the final model. In particular, clutch size was retained as a covariate in models analysing OXY concentrations, and on day 12, total sampling time was fitted as a covariate in the model for ROM concentrations.

All statistical analyses were performed in R statistical freeware R-3.3.3 [79]. Principal component analyses were performed using the ‘*prcomp*’ package. Statistical models were conducted using the ‘l*me4*’ and ‘*arm*’ packages in a Bayesian framework with non-informative priors. We assumed a Poisson error distribution for the generalized linear models and a Gaussian error distribution for the linear mixed-effect models. In all cases, residuals were checked visually for the model fit. Whenever necessary, response variables were transformed (details on transformations are provided in the tables). We mean-centered all covariates (i.e., mean value = 0, standard deviation = 1) because covariates differed in their scales of magnitude. We based model structure on the study question and the biology of the species rather than on model selection [80]. We subsequently used the ‘sim’ function to simulate values from the posterior distributions of model parameters. From 10,000 simulations, we extracted the 95% Bayesian credible interval (CrI) around the mean [77] and assessed statistical support by obtaining the posterior distribution of each parameter. CrIs provide more valuable information than p-values, such as the uncertainty around the estimates. We use the term ‘statistically meaningful’ when the estimated effect differed from zero with a posterior probability higher than 0.95. A threshold of 5% is equivalent to the significance level in a frequentist framework (for further details on statistical inference, see [80].

## Supplementary Information

**Additional file 1.** Principal component analysis of 31 yolk components measured in the fourth egg of 69 wild great tit clutches.

**Additional file 2.** Results of linear models and linear mixed-effects models to test for relationships between yolk composition and fitness traits.

**Additional file 3.** Results of linear mixed-effects models to test for relationships between yolk composition and phenotypic traits on nestling day 6.

**Additional file 4.** Results of linear mixed-effects models to test for relationships between yolk composition and phenotypic traits on day 12.

**Additional file 5.** Results of linear mixed-effects models used to test for relationships between nestling tarsus length on days 6 and 12 and survival in the nest.

**Additional file 6.** Methods.

**Additional file 7.** Linear models testing for differences in yolk composition between years.

## Data Availability

The datasets and code supporting this article are available from the Dryad Digital Repository at https://datadryad.org/stash/share/IcnE-oztb_zA3DqBchqfZMcL9Ly_dYzl0HkyMxZLIX0.
