# Supplementary material for: Natural variation in yolk fatty acids, but not androgens, predicts offspring fitness in a wild bird
Source: Front Zool. 2021 Aug 5;18:38. doi: 10.1186/s12983-021-00422-z (PMC8340462; doi:10.1186/s12983-021-00422-z)
Supplement: Supplementary file 1 — Additional file 1. Principal component analysis of 31 yolk components measured in the fourth egg of 69 wild great tit clutches. [file 12983_2021_422_MOESM1_ESM.docx]

Additional file 1. Principal component analysis (PCA) of 31 yolk components measured in the fourth egg of 69 wild great tit clutches. The yolk components with highest loadings in a given PC are presented in bold. Note: fatty acids lacking a common name are listed using their systematic names.

| Yolk components | Yolk groups | PC1 | PC2 | PC3 |
| --- | --- | --- | --- | --- |
| Eigenvectors | | | | |
| Androstenedione | Steroid  hormones | -0.13 | 0.13 | **0.28** |
| 5α-dihydrotestosterone |  | 0.02 | 0.07 | **0.43** |
| Testosterone |  | -0.00 | 0.07 | **0.30** |
| Corticosterone |  | 0.11 | 0.03 | 0.15 |
| Vitamin E | Antioxidants | **-0.21** | 0.02 | 0.05 |
| Lutein |  | 0.14 | -0.14 | **0.32** |
| Zeaxanthin |  | 0.15 | -0.03 | **0.31** |
| Lauric acid (12:0) | Saturated fatty acids  (SFA) | 0.04 | 0.14 | 0.16 |
| Myristic acid (14:0) |  | 0.10 | 0.18 | 0.10 |
| Pentadecanoic acid (15:0) |  | -0.02 | **0.32** | 0.14 |
| Palmitic acid (16:0) |  | 0.09 | **0.40** | 0.04 |
| Margaric acid (17:0) |  | -0.13 | **0.20** | 0.13 |
| Stearic acid (18:0) |  | -0.18 | **0.28** | -0.19 |
| Oleic acid (18:1n-9) | Monounsaturated  fatty acids  (MUFA) | 0.14 | **0.28** | **-0.28** |
| Hexadecenoic acid (16:1n-9) |  | 0.05 | **0.24** | **-0.31** |
| Palmitoleic acid (16:1n-7) |  | 0.11 | **0.20** | -0.03 |
| *cis*-Vaccenic acid (18:1n-7) |  | -0.18 | **0.24** | -0.03 |
| Eicosenoic acid (20:1n-9) |  | **-0.22** | 0.05 | -0.11 |
| α-Linolenic acid (18:3n-3) | ω-3 Polyunsaturated fatty acids  (ω-3 PUFA) | 0.18 | **0.28** | 0.13 |
| Eicosapentaenoic acid (20:5n-3) |  | 0.01 | **0.28** | 0.13 |
| Docosapentaenoic acid (22:5n-3) |  | 0.09 | **0.25** | -0.08 |
| Docosahexaenoic acid (22:6n-3) |  | -0.02 | **0.25** | 0.03 |
| γ-linolenic acid (18:3n-6) | ω-6 Polyunsaturated fatty acids  (ω-6 PUFA) | **-0.27** | 0.08 | 0.03 |
| Hexadecadienoic acid (16:2n-6) |  | **-0.29** | 0.03 | 0.08 |
| Linoleic acid (18:2n-6) |  | **-0.29** | 0.08 | 0.01 |
| Pinolenic acid (18:3n-6) |  | **-0.29** | -0.01 | 0.13 |
| Eicosadienoic acid (20:2n-6) |  | **-0.28** | -0.08 | 0.01 |
| dihomo- γ -Linolenic acid (20:3n-6) |  | **-0.28** | -0.02 | 0.08 |
| Arachidonic acid (20:4n-6) |  | **-0.22** | 0.07 | -0.17 |
| Adrenic acid (22:4n-6) |  | **-0.23** | 0.02 | -0.07 |
| Eigenvalue | | | | |
| Standard deviation | | 3.28 | 2.09 | 1.69 |
| % Total variance | | 34.69 | 14.08 | 9.31 |
| % Cumulative variance | | 34.69 | 48.77 | 58.08 |
