# Supplementary material for: Natural variation in yolk fatty acids, but not androgens, predicts offspring fitness in a wild bird
Source: Front Zool. 2021 Aug 5;18:38. doi: 10.1186/s12983-021-00422-z (PMC8340462; doi:10.1186/s12983-021-00422-z)
Supplement: Supplementary file 2 — Additional file 2. Results of linear models and linear mixed-effects models to test for relationships between yolk composition and fitness traits. [file 12983_2021_422_MOESM2_ESM.docx]

Additional file 2. Results of linear models and linear mixed-effects models to test for relationships between yolk composition and fitness traits. PC1, PC2, PC3, date and clutch size were included as covariates. All covariates were mean-centered. Nest ID was included as a random factor in the linear mixed-effect models. We present fixed (β) and random (σ^2^) parameters with their 95% credible intervals (CrIs) in brackets. Fixed factors with a statistically meaningful effect (i.e., if the mean difference between compared estimates was higher than 0.95) are presented in bold.

^a^ PC1 was mainly represented by low concentrations of vitamin E (α - tocopherol) and ω-6 polyunsaturated fatty acids (PUFAs).

^b^ PC2 was mainly represented by high concentrations of saturated (SFA), mono-unsaturated (MUFA) and all ω-3 PUFAs.

^c^ PC3 was mainly represented by high concentrations of androgens (androstenedione, 5α-dihydrotestosterone and testosterone) and carotenoids (lutein and zeaxanthin).

^d^ Date when the fourth egg was collected.

|  | Hatchling number | Fledgling number | Fledgling mass corrected for clutch size | Fledgling tarsus corrected for clutch size |
| --- | --- | --- | --- | --- |
| Fixed factors β (95% CrI) | | | | |
| Intercept | 1.46  (1.35; 1.57) | 0.73  (0.55; 0.91) | 0.03  (-0.51; 0.56) | 0.01  (-0.19; 0.22) |
| PC1^a^ | -0.03  (-0.15; 0.08) | **0.19**  **(0.03; 0.34)** | -0.19  (-0.78; 0.39) | -0.09  (-0.31; 0.14) |
| PC2^b^ | **0.10**  **(-0.01; 0.21)** | **0.29**  **(0.17; 0.43)** | 0.13  (-0.47; 0.73) | -0.18  (-0.05; 0.42) |
| PC3^c^ | -0.08  (-0.20; 0.06) | 0.02  (-0.16; 0.19) | 0.10  (-0.49; 0.69) | 0.05  (-0.18; 0.28) |
| Date^d^ | -0.01  (-0.15; 0.13) | **-0.46**  **(-0.75; -0.15)** | -0.08  (-0.55; 0.38) | 0.02  (-0.17; 0.21) |
| Clutch size | 0.07  (-0.06; 0.19) | **-0.21**  **(-0.40; -0.02)** | - | - |
| Random factors σ^2^ (95% CrI) | | | | |
| Nest ID | - | - | 2.25  (1.69; 2.94) | 0.27  (0.18; 0.38) |
| Residual variance | - | - | 1.57  (1.26; 1.97) | 0.52  (0.41; 0.65) |
