# Supplementary material for: Natural variation in yolk fatty acids, but not androgens, predicts offspring fitness in a wild bird
Source: Front Zool. 2021 Aug 5;18:38. doi: 10.1186/s12983-021-00422-z (PMC8340462; doi:10.1186/s12983-021-00422-z)
Supplement: Supplementary file 3 — Additional file 3. Results of linear mixed-effects models to test for relationships between yolk composition and phenotypic traits on nestling day 6. [file 12983_2021_422_MOESM3_ESM.docx]

Additional file 3. Results of linear mixed-effects models to test for relationships between yolk composition and phenotypic traits on nestling day 6. PC1, PC2, PC3, date and clutch size were included as covariates. All covariates were mean-centered. Nest ID was included as a random factor in the linear mixed-effect models. We present fixed (β) and random (σ^2^) parameters with their 95% credible intervals (CrIs) in brackets. Fixed factors with a statistically meaningful effect (i.e., if the mean difference between compared estimates was higher than 0.95) are presented in bold.

^a^ PC1 was mainly represented by low concentrations of vitamin E (α - tocopherol) and ω-6 polyunsaturated fatty acids (PUFAs).

^b^ PC2 was mainly represented by high concentrations of saturated (SFAs), mono-unsaturated (MUFAs) and all ω-3 PUFAs.

^c^ PC3 was mainly represented by high concentrations of androgens (androstenedione, 5α-dihydrotestosterone and testosterone) and carotenoids (lutein and zeaxanthin).

^d^ Date when the fourth egg was collected.

^e^ Non-enzymatic antioxidant measured in plasma.

^f^ Oxidative damage compounds measured in plasma.

|  | OXY ^e^ | ROMs ^f^ | Nestling  mass corrected for clutch size | Nestling  tarsus corrected for clutch size |
| --- | --- | --- | --- | --- |
| Fixed factors β (95% CrI) | | | | |
| Intercept | 223.17  (204.08; 241.92) | 1.12  (1.00; 1.25) | -0.02  (-0.39; 0.35) | -0.03  (-0.31; 0.26) |
| PC1 ^a^ | -11.40  (-30.96; 8.27) | -0.02  (-0.15; 0.11) | -0.11  (-0.48; 0.26) | -0.11  (-0.38; 0.17) |
| PC2 ^b^ | -4.32  (-25.94; 17.56) | 0.05  (-0.08; 0.19) | 0.20  (-0.15; 0.56) | **0.24**  **(-0.01; 0.50)** |
| PC3 ^c^ | **-17.77**  **(-37.40; 1.93)** | 0.05  (-0.08; 0.18) | 0.07  (-0.35; 0.47) | 0.07  (-0.22; -.37) |
| Date ^d^ | -7.23  (-27.36; 13.12) | -0.07  (-0.21; 0.05) | -0.12  (-0.49; 0.25) | -0.15  (-0.43; 0.13) |
| Clutch size | **-19.59**  **(-39.91; -0.01)** | - | - | - |
| Random factors σ^2^ (95% CrI) | | | | |
| Nest ID | 3311.55  (2400.79; 4389.16) | 0.12  (0.08; 0.16) | 1.57  (1.19; 2.02) | 0.77  (0.56; 1.02) |
| Residual variance | 3749.83  (3051.48; 4623.26) | 0.26  (0.21; 0.32) | 1.65  (1.38; 1.97) | 1.34  (1.18; 1.59) |
