# Supplementary material for: Natural variation in yolk fatty acids, but not androgens, predicts offspring fitness in a wild bird
Source: Front Zool. 2021 Aug 5;18:38. doi: 10.1186/s12983-021-00422-z (PMC8340462; doi:10.1186/s12983-021-00422-z)
Supplement: Supplementary file 4 — Additional file 4. Results of linear mixed-effects models to test for relationships between yolk composition and phenotypic traits on day 12. [file 12983_2021_422_MOESM4_ESM.docx]

Additional file 4. Results of linear mixed-effects models to test for relationships between yolk composition and phenotypic traits on day 12. PC1, PC2, PC3, date and clutch size were included as covariates. All covariates were mean-centered. Nest ID was included as a random factor in the linear mixed-effect models. We present fixed (β) and random (σ^2^) parameters with their 95% credible intervals (CrIs) in brackets. Fixed factors with a statistically meaningful effect (i.e., if the mean difference between compared estimates is higher than 0.95) are presented in bold.

^a^ PC1 was mainly represented by low concentrations of vitamin E (α - tocopherol) and ω-6 polyunsaturated fatty acids (PUFAs).

^b^ PC2 was mainly represented by high concentrations of saturated fattyb acids (SFAs), mono-unsaturated (MUFAs) and all ω-3 PUFAs.

^c^ PC3 was mainly represented by high concentrations of androgens (androstenedione, 5α-dihydrotestosterone and testosterone) and carotenoids (lutein and zeaxanthin).

^d^ Date when the fourth egg was collected.

^e^ Time since we arrived at the nest until we finished taking blood from each individual

^f^ Non-enzymatic antioxidant measured in plasma.

^g^ Enzymatic antioxidant measured in red blood cells.

GPX was square-root transformed.

^h^ Oxidative damage compounds measured in plasma.

ROMs was log10 transformed.

|  | OXY ^f^ | GPX ^g^ | ROMs ^h^ | Nestling  mass corrected for clutch size | Nestling  tarsus corrected for clutch size |
| --- | --- | --- | --- | --- | --- |
| Fixed factors β (95% CrI) | | | | | |
| Intercept | 221.58  (208.60; 234.86) | 1.54  (1.23; 1.84) | 0.27  (0.15; 0.38) | 0.08  (-0.58; 0.75) | 0.03  (-0.30; 0.36) |
| PC1 ^a^ | 1.30  (-12.47; 15.36) | **0.37**  **(0.06; 0.68)** | **-0.12**  **(-0.24; 0.00)** | -0.30  (-1.01; 0.41) | -0.19  (-0.54; 0.16) |
| PC2 ^b^ | **-11.81**  **(-26.54; 2.85)** | -0.09  (-0.40; -0.21) | 0.07  (-0.04; 0.19) | 0.04  (-0.53; 0.61) | 0.12  (-0.17; 0.39) |
| PC3 ^c^ | **-11.43**  **(-25.02; 1.87)** | -0.04  (-0.35; 0.26) | -0.01  (-0.13; 0.11) | -0.34  (-1.07; 0.40) | -0.05  (-0.43; 0.33) |
| Date ^d^ | 1.56  (-12.67; 15.55) | 0.21  (-0.10; 0.51) | -0.01  (-0.13; 0.11) | -0.36  (-0.92; 0.21) | -0.25  (-0.54; 0.05) |
| Clutch size | -9.25  (-24.53; 5.81) | - | - | - | - |
| Total sampling time ^e^ | - | - | **-0.08**  **(-0.16; 0.00)** | - | - |
| Random factors σ^2^ (95% CrI) | | | | | |
| Nest ID | 519.85  (295.15; 820.63) | 0.39  (0.24; 0.59) | 0.07  (0.05; 0.11) | 3.22  (2.44; 4.27) | 0.76  (0.56; 1.03) |
| Residual variance | 2659.98  (1956.52; 3611.16) | 1.17  (0.87; 1.57) | 0.12  (0.09; 0.17) | 1.87  (1.49; 2.33) | 0.71  (0.57; 0.89) |
