# Supplementary material for: Natural variation in yolk fatty acids, but not androgens, predicts offspring fitness in a wild bird
Source: Front Zool. 2021 Aug 5;18:38. doi: 10.1186/s12983-021-00422-z (PMC8340462; doi:10.1186/s12983-021-00422-z)
Supplement: Supplementary file 5 — Additional file 5. Results of linear mixed-effects models used to test for relationships between nestling tarsus length on days 6 and 12 and survival in the nest. [file 12983_2021_422_MOESM5_ESM.docx]

Additional file 5. Results of linear mixed-effects models used to test for relationships between nestling tarsus length on days 6 and 12 and survival in the nest. Tarsus length, clutch size and date were included as covariates. All covariates were mean-centered. Nest ID was included as a random factor in the linear mixed-effect models. We present fixed (β) and random (σ^2^) parameters with their 95% credible intervals (CrIs) in brackets. Fixed factors with a statistically meaningful effect (i.e., if the mean difference between compared estimates is higher than 0.95) are presented in bold.

|  | Survived (day 6 to day 12) ^c^ | Survived (day 12 to day 15) ^d^ |
| --- | --- | --- |
| Fixed factors β (95% CrI) | | |
| Intercept | 7.82  (2.85; 12.79) | 4.87  (2.83; 6.89) |
| Tarsus ^a^ | **1.86**  **(0.81; 2.94)** | **1.35**  **(0.34; 2.35)** |
| Clutch size | 0.32  (-4.09; 4.69) | -0.68  (-2.15; 0.78) |
| Date ^b^ | -0.74  (-4.14; 2.64) | **-0.75**  **(1.58; 0.06)** |
| Random factors σ^2^ (95% CrI) | | |
| Nest ID | 116.66  (70.74; 175;13) | - |
| Residual variance | 0.99  (0.99; 0.99) | - |

^a^ Nestling tarsus length measured on day 6 and day 12 (first and second models, respectively).

^b^ Date when the fourth egg was collected.

^c^ Nestlings measured on day 6 (i.e., from early development) that survived to day 12 (i.e., period when chicks experienced exponential growth).

^d^ Nestlings measured on day 12 that survived to day 15 (i.e., when nestlings were about to fledge).
