# Supplementary material for: Natural variation in yolk fatty acids, but not androgens, predicts offspring fitness in a wild bird
Source: Front Zool. 2021 Aug 5;18:38. doi: 10.1186/s12983-021-00422-z (PMC8340462; doi:10.1186/s12983-021-00422-z)
Supplement: Supplementary file 6 — Additional file 6. Methods. [file 12983_2021_422_MOESM6_ESM.docx]

Additional file 6

Methods

*Environmental conditions*

We collected data on ambient temperature and rainfall during both breeding seasons, since these key environmental factors are known to affect fitness in songbirds (Visser et al., 1998; Öberg et al., 2015). We recorded ambient temperature (°C) every hour by placing 12 i-buttons (DS9093A+ Thermochron iButton) in different locations in the forest, whilst we obtained information on total rainfall (mm) from a meteorological station less than 5 km from the field site (Oberpfaffenhofen, Germany: 48° 05’ N, 11 ° 16' E, 583 m above sea level).

*Yolk analyses*

*Steroid hormones*

We measured yolk steroid hormones following Mentesana et al. (2019). To separate and measure steroid hormones (androstenedione, 5α-dihydrotestosterone, testosterone, and corticosterone) we followed the method described by Wingfield and Farner (1976), as modified by Goymann et al. (2008) and with additional adjustments for the measurement of egg yolk following Schwabl (1993). On average, we transferred 50 µl of the yolk/water emulsion to 16 × 100 glass test tubes. Along with the samples, we also prepared two blanks containing 300 µl distilled water and three positive controls containing 100 µl stripped chicken plasma pools. We added distilled water to all tubes to have the same final volume (300 µl). Then, we added 10 µl tritiated steroid (at 1500 dpm; PerkinElmer, MA, USA) of all steroid hormones to be measured to all tubes except the blanks to estimate extraction efficiency. Next, we added 4 ml of diethyl ether to each sample. After overnight equilibration, we centrifuged the samples and collected the supernatant which we dried under a nitrogen stream in a water bath at 40°C. We then subjected each sample to a second extraction by adding 2 ml dichloromethane. We re-suspended the dried supernatant in 1 ml 99% ethanol. After an overnight reconstitution, we centrifuged the extracts, collected the supernatant, again dried under a nitrogen stream in a water bath at 40°C, and reconstituted in isooctane with 2% ethyl acetate. Further, we separated steroid hormones via diatomaceous earth column chromatography. The columns had the following specifications: length: 29.20 cm, diameter: 0.77 cm, diameter of the tip: 0.35 cm, thickness of the glass: 0.10 cm, volume: 5 ml (1/10), flat ending, material: Pyrex glass, TD-ED 20 ºC. We used celite 521 from Sigma-Aldrich (Cat.: 22, 179-1, CAS 61790-53-2). We eluted fractions containing different steroids by mixing ethyl acetate (EtAc) with isooctane at increasing concentrations (2, 10, 25, and 45% EtAc for androstenedione, 5α-dihydrotestosterone, testosterone and corticosterone, respectively). We collected each eluted fraction in 12x75 mm glass tubes and evaporated their content under a nitrogen stream. We re-dissolved androgens (androstenedione, 5α-dihydrotestosterone, and testosterone) in 300 µl phosphate-buffered saline. We used 80 µl of the resuspended fraction to estimate individual extraction recoveries. Recoveries for the three sets of column chromatographies for androgens were within the range previously reported for great tits (Tschirren et al. 2004; Groothuis et al. 2008; Lessels et al. 2016; Mentesana et al. 2019) (mean ± SD): androstenedione = 80.33 ± 4.93%, 5α-dihydrotestosterone = 56.33 ± 4.04%, testosterone = 66 ± 15.62%. We quantified androgen concentrations using radio-immunoassays. For this, we used the following polyclonal antibodies: AN6-22 for androstenedione, DT3-351 for 5α-dihydrotestosterone and T3-125 for testosterone (all Esoterix Endocrinology, CA, USA). The lower detection limit was at 7.1 pg/ml for andro¬stenedione, 6.6 pg/ml for 5α-dihydrotestosterone and 3.9 pg/ml for testosterone. Blanks were always below detection limits. We dried and then dissolved the corticosterone fractions of the samples in 350 µl assay buffer. We used an aliquot of 80 μl to estimate individual extraction recoveries (mean ± SD recoveries were 55 ± 5.29%).

*Antioxidants*

We simultaneously extracted vitamin E (α-tocopherol), lutein and zeaxanthin following Mentesana et al. (2019). For this, to 20 mg yolk we added 200 µl of acetone that contained an internal standard: 600 µM retinyl acetate and 1 mM tocopheryl acetate (Sigma-Aldrich, Stockholm, Sweden). We then vortexed the samples and left them to equilibrate overnight at –80°C. The following day, we added 200 µl tert-butyl methyl ether to each sample and vortexed them. We then centrifuged samples at 10°C for 5 min (13000 rpm). Next, we transferred the supernatant to a new tube and dried it under nitrogen gas. We washed the samples twice with 200 µl acetone, followed by vortexing and centrifugation. We removed the supernatant to a new tube between the acetone washes. Again, we dried the samples under nitrogen gas. We dissolved the residue in 100 µl methanol-acetonitrile (30:70). We determined the amount of α-tocopherol, lutein and zeaxanthin by high performance liquid chromatography (HPLC) with the following specifications: column Phenomenex Syndergi 4u Hydro-RP 80A, 250.30 mm + 4.2 mm guard column, isocratic 20% MeOH, 80% AcCN, 12 min, 1.2 ml min–1, oven 40°C, injection 5 µl, UV 450 nm, FL ex: 290 nm, em: 325 nm.

*Fatty acids*

We extracted fatty acids following the methods described by Eikenaar et al. (2017) and Mentesana et al. (2019). We extracted total lipids from approximately 5 mg of yolk using 50 µl chloroform and methanol (2:1 v/v) mixed with 16.65 µg of the internal standard methyl *cis*-10-heptadecenoate (purity >99%; Aldrich). We then dried the samples under N_2_ and carried out base methanolysis (using 100 mL 0.5 M KOH/Me; reaction proceeded for 1h at 40 °C and terminated using 100 mL 0.5 M HCl/Me) to transform the fatty acids into the corresponding fatty acid methyl esters (FAMEs). We then extracted the FAMEs using heptane (> 99%; VWR Prolabo). The extracts were washed with deionized H_2_O, and residual water was removed using anhydrous sodium sulfate. We analysed the extracts using an Agilent 5975 mass spectrometer coupled to an Agilent 6890 gas chromatograph with an HP-INNOWax PEG column (Agilent) with the following specifications: 30 m, 0.25 mm i.d., 0.25 mm film thickness. We performed analyses and quantification of chromatograms using ChemStation software (Agilent). We identified FAMEs by comparing mass spectra and retention times with those of synthetic standards (Supelco 37-Component FAME Mix, Sigma-Aldrich).

*Oxidative biomarkers measurements*

*Non-enzymatic antioxidant (OXY)*

We measured non-enzymatic antioxidants using the OXY-Adsorbent test (Diacron, Grosseto, Italy) following Costantini et al. (2006). For this, we diluted plasma at a 2:50 ratio in distilled water followed by vortexing. We then took 2 µl of the diluted sample, pipetted samples in duplicate into plate wells and added an aliquot of 200 µl of HOCl to each well. Along with the samples, we also prepared a standard (i.e., only HOCl/ml) and a blank (i.e., only distilled water). We sealed the plate, shook it for 30 secs and then incubated it for 10 min at 37°C. At the end of the incubation, we added 2 µl of the chromogen N, N-diethyl-p-phenylenediamine. We measured the intensity of the coloured complex, which is inversely related to the antioxidant power, with a spectrophotometer (Thermo Scientific Multiskan GO; Thermo Fisher Scientific Corporation) at 546 nm. We calculated final non-enzymatic antioxidant concentrations using the formula: (Absorbance blank – Absorbance sample) / (Absorbance blank – Absorbance standard) x std (350 µlmol HOCl/ml)

*Enzymatic antioxidant (GPX)*

We measured the activity of the enzymatic antioxidant glutathione peroxidase (GPX) in red blood cells using the Ransel assay (Randox Laboratories, Crumlin, UK) following Costantini et al. (2011). For this, we diluted red blood cells at 1:40 ratio in a diluting agent provided with the assay (i.e., R3). We then centrifuged the solutions at 3000 rpm for 1 min. Next, we pipetted 200 µl of the R1 reagent and added 4 µl of the solutions to each plate well. We also added 8 µl of the R2 into each well. Along with the samples, we also prepared a standard (i.e., only HOCl/ml) and a blank (i.e., only distilled water). We read the absorbance three times using the Thermo Scientific Multiskan GO (Thermo Fisher Scientific Corporation) at 340 nm for 1 min. The temperature inside the plate reader was maintained at 37°C. We calculated final GPX concentrations using the formula: ((Absorbance sample – Absorbance blank) * 40) x 8412) / 1000

*Reactive oxygen species (ROMs)*

The concentration of organic hydroperoxides (ROMs) was measured using the d-ROMs test (Diacron, Grosseto, Italy) following Costantini et al. (2006). For this, we diluted 2 µl of plasma in 100 µl of a solution made by mixing two agents provided with the assay (i.e., R1 and R2). We pipetted each sample in duplicate into plate wells. Along with the samples, we also prepared a standard (i.e., only the solution of acetic acid/sodium acetate buﬀer and N, N-diethyl-p-phenylenediamine) and a blank (i.e., only distilled water). We then sealed the plate, shook it for 3 secs and then incubated it for 90 min at 37°C. Next, we measured the intensity of the coloured complex, which is proportional to the oxidant capacity, with a spectrophotometer (Thermo Scientific Multiskan GO; Thermo Fisher Scientific Corporation) at 546 nm. We calculated final ROMs concentrations from standard curves made with the above-mentioned standard.

*Statistical analyses*

*Principal Component Analysis (PCA)*

To test for interactive effect of group yolk components, we ran a principal component analysis (PCA) including all 31 yolk components analysed. PCA was selected over other multivariate analyses because it is a data reduction method that can be used to characterize the correlation structure of a set of related variables (Budaev 2010; Paliy & Shankar 2016). For each principal component, we only considered the variables that had absolute values of loadings higher than 0.2. Because the minimum cut-off is disputed, we decided on that value based on our research question and on the correlation between yolk components (Mentesana et al. unpublished).

*Relationship between yolk components and fitness proxies*

In the models for hatchling and fledgling number, clutch size was included as a covariate. Two clutches were excluded from the analyses: one clutch was depredated during the incubation period and was excluded from the models using hatchling and fledgling number as a response variable. Another clutch was excluded from the model using fledgling number as a response variable after inspection of the residuals (i.e., Cook’s distance plot).

Environmental variables such as mean ambient temperature and mean rainfall during incubation were included in the model for hatchling number, whilst mean ambient temperature and mean rainfall during the nestling period were included in those models for fledgling number and body condition. Time of capture was also initially included in the models for nestling body condition. However, since neither the environmental parameters nor the time of capture explained the response variables, we excluded these from the final models to avoid overparameterization.

*Relationship between egg components and offspring physiological condition*

To study the relationship between egg components and the physiological condition of individual nestlings from a given brood we initially included other covariates in the model based on their biological relevance to the study question. These were clutch size, time of capture, ambient temperature at time of capture and total sampling time (i.e., time from arrival at the nest until the end of blood sampling for each individual). But because of sample size constraints, only variables that had an effect on the response variables were retained in the final model.

*Complementary statistical analyses for the Discussion*

*Nestling size and survival*

To understand whether nestling size (tarsus length) was associated with nestling survival, we ran linear mixed-effect models with a Poisson distribution. We first fitted survival from day 6 to day 12 (i.e., from early development to the period when chicks showed exponential growth) as the response variable. Next, we fitted survival from day 12 to day 15 (i.e., until the time when nestlings were about to fledge). Tarsus length (either on day 6 or 12), clutch size and date of capture were included as covariates, and nest ID was included as a random factor.

Results from linear mixed-effect models were considered to be statistically meaningful when the posterior probability of the mean difference between compared estimates was higher than 0.95. Further information on our statistical approach can be found in Materials and Methods. Results are presented in the Supplementary Table 6.

**References**

Budaev SV. (2010). Using principal components and factor analysis in animal behaviour research: caveats and guidelines. Ethology. 116, 472–480. (doi: [10.1111/j.1439-0310.2010.01758.x](https://doi.org/10.1111/j.1439-0310.2010.01758.x)).

Costantini D, Casagrande S, De Filippis S, Brambilla G, Fanfani A, Tagliavini J, Dell’Omo G. (2006). Correlates of oxidative stress in wild kestrel nestlings (*Falco tinnunculus*). J. Comp. Physiol. B. 176, 329–337. (doi: 10.1007/s00360-005-0055-6).

Costantini D, Monaghan P, Metcalfe NB. (2011). Biochemical integration of blood redox state in captive zebra finches (Taeniopygia guttata). J. Exp. Biol. 214, 1148-1152. (doi: 10.1242/jeb.053496).

Eikenaar C, Källstig E, Andersson MN, Herrera-Dueñas A, Isaksson C. (2017). Oxidative challenges of avian migration: a comparative field study on a partial migrant. Physiol. Biochem. Zool. 90, 223–229. (doi: 10.1086/689191).

Goymann W, Wittenzellner A, Schwabl I, Makomba M. (2008). Progesterone modulates aggression in sex-role reversed female African black coucals. Proc. R. Soc. B. 275, 1053–1060. (doi: doi:10.1098/rspb.2007.1707).

Groothuis TGG, Carere C, Lipar J, Drent PJ, Schwabl H. (2008). Selection on personality in a songbird affects maternal hormone levels tuned to its effect on timing of reproduction. Biol. Lett. 4: 465– 467. (doi: 10.1098/rsbl.2008.0258).

Lessells CM, Ruuskanen S, Schwabl H. (2016). Yolk steroids in great tit Parus major eggs: variation and covariation between hormones and with environmental and parental factors. Behav. Ecol. Sociobiol. 70, 843–856. (doi: 10.1007/s00265-016-2107-1).

Mentesana L, Isaksson C, Goymann W, Andersson MN, Trappschuh M, Hau M. (2019). Female variation in allocation of steroid hormones, antioxidants and fatty acids: a multilevel analysis in a wild passerine bird. J. Avian Biol. 50(1), e01859. (doi: 10.1111/jav.01859).

Öberg M, Arlt D, Pärt T, Laugen AT, Eggers S, Low M. (2015) Rainfall during parental care reduces reproductive and survival components of fitness in a passerine bird. *Evol. Ecol*. 5, 345– 356. (doi: 10.1002/ece3.1345).

Paliy O, Shankar V. (2016). Application of multivariate statistical techniques in microbial ecology. *Mol. Ecol.* 25, 1032–1057. (doi: 10.1111/mec.13536).

Schwabl H. (1993). Yolk is a source of maternal testosterone for developing birds. Proc. Natl. Acad. Sci. 90, 11446–11450. (doi: doi:10.1073/pnas.90.24.11446).

Tschirren B, Richner H, Schwabl H. (2004). Ectoparasite–modulated deposition of maternal androgens in great tit eggs. Proc. R. Soc. B. 271, 1371-1375. (doi: 10.1098/rspb.2004.2730).

Visser ME, Noordwijk AJ, Tinbergen J, Lessells CM. (1998). Warmer springs lead to mistimed reproduction in great tits (*Parus major*). *Proc. R. Soc. B.* 265: 1867–1870. (doi: 10.1098/rspb.1998.0514).

Wingfield JC, Farner DS. (1976). Avian endocrinology: field investigations and methods. Condor. 78, 570–573. (doi: doi:10.2307/1367117).
