# Supplementary material for: Natural variation in yolk fatty acids, but not androgens, predicts offspring fitness in a wild bird
Source: Front Zool. 2021 Aug 5;18:38. doi: 10.1186/s12983-021-00422-z (PMC8340462; doi:10.1186/s12983-021-00422-z)
Supplement: Supplementary file 7 — Additional file 7. Linear models testing for differences in yolk composition between years. [file 12983_2021_422_MOESM7_ESM.docx]

Additional file 7. Linear models testing for differences in yolk composition (as represented by PC1, PC2 and PC3) between years. Year (2015 or 2016) was fitted as a fixed factor. We present fixed (β) parameters with their 95% credible intervals (CrIs) in brackets. Fixed factors with a statistically meaningful effect (i.e., if the mean difference between compared estimates is higher than 0.95) are presented in bold.

|  | PC1 ^a^ | PC2 ^b^ | PC3 ^c^ |
| --- | --- | --- | --- |
| Fixed factors β (95% CrI) | | | |
| Intercept | 3.41  (2.07; 4.78) | -0.02  (-1.08; 1.02) | -1.07  (-1.88; -0.25) |
| Year | **-4.46**  **(-6.01; -2.93)** | 0.03  (-1.19; 1.22) | **1.38**  **(0.47; 2.29)** |

^a^ PC1 was mainly represented by low concentrations of vitamin E (α - tocopherol) and ω-6 polyunsaturated fatty acids (PUFAs).

^b^ PC2 was mainly represented by high concentrations of saturated (SFAs), mono-unsaturated (MUFAs) and all ω-3 PUFAs.

^c^ PC3 was mainly represented by high concentrations of androgens (androstenedione, 5α-dihydrotestosterone and testosterone) and carotenoids (lutein and zeaxanthin).
